# CMV retinitis: the diagnostic challenges and long-term outcomes. The experience of tertiary eye center in Saudi Arabia

**DOI:** 10.1186/s12348-026-00572-3

**Published:** 2026-03-11

**Authors:** Abdulrahman AlZaid, Abdulrahman Khan, Hassan Al-Dhibi, Moustafa S. Magliyah

**Affiliations:** 1 Vitreoretinal Division, King Khaled Eye Specialist Hospital, Riyadh, Kingdom of Saudi Arabia; 2 Vitreoretinal and Uveitis Division, King Khaled Eye Specialist Hospital, PO Box 7141, Riyadh, 11462 Saudi Arabia

**Keywords:** CMV retinitis, HIV, Retinal detachment, Valganciclovir, Immunocompromised status

## Abstract

**Purpose:**

To describe the clinical features, complications, and long-term visual outcomes of cytomegalovirus (CMV) retinitis in patients presenting to a tertiary eye center.

**Methods:**

A retrospective chart review of patients who were diagnosed with CMV retinitis between 2014 and 2024.

**Results:**

Twelve eyes of 8 patients were included. Five patients (62.5%) were males and 3 (37.5%) were females. All patients were immunocompromised. Four patients (40%) were on immunosuppressive medications after renal transplantations for chronic renal failure (CRF), three patients had human immunodeficiency virus (HIV) infections, and one patient had a congenital immunodeficiency disease. The baseline best-corrected visual acuity (BCVA) was 0.8 ± 0.9 (Snellen = 20/125). Seven eyes (58.3%) had a hemorrhagic type of retinitis, and 5 eyes had granular retinitis (41.7%). Vitritis was found only in 2 eyes (16.7%), vasculitis was found in 3 eyes (25%), and occlusive vasculitis was found in 2 eyes (16.7%), and all of these features were present in patients who were non-HIV infected. The mean BCVA on the last visit was 0.9 ± 1.2 (Snellen = 20/160). Visual threatening complications included macular atrophy, optic disc pallor, rhegmatogenous retinal detachment (RRD), and NVG.

**Conclusion:**

The clinical picture of CMV retinitis is better related to the level of immunity than the classification of HIV vs. non-HIV related. Signs of inflammatory response were absent in HIV-infected patients.

**Supplementary Information:**

The online version contains supplementary material available at 10.1186/s12348-026-00572-3.

## Introduction

Cytomegalovirus (CMV) is an opportunistic virus that belongs to the Herpesviridae family, which are double-stranded deoxyribonucleic acid (DNA) viruses that vary in size between 120 and 160 nm. CMV has a prevalence of about 50–88% in the general population [1, 2]. CMV retinitis is a clinical term that describes the reactivation of CMV, leading to prominent retinal inflammation in the absence of anterior chamber reaction or vitritis as a result of compromised immune status [1]. Before the era of human immunodeficiency virus (HIV), CMV retinitis was observed mainly in iatrogenically immunosuppressed patients, including transplant recipients and patients receiving chemotherapy [3–7]. After the emergence of HIV, CMV retinitis was commonly found in patients with HIV infection and low CD4 counts [8–10]. In addition, patients who have undergone organ transplantations are predisposed to CMV retinitis [11–16]. The development of Highly Active Antiretroviral Therapy (HAART), which controls the replication of HIV and induces immune restoration, in addition to advances in immunosuppressive treatment options after organ transplantation, allowed for a better understanding of the clinical picture and outcomes of CMV retinitis [10, 13, 14]. Two clinical pictures of CMV retinitis have been described: a fulminant picture that presents with confluent necrotizing retinitis and dispersed retinal hemorrhages, and an indolent picture characterized by a granular retinitis with few or absent hemorrhages. In a previous study, which compared the clinical pictures of CMV retinitis in HIV infected and non-HIV-infected patients, the presence of vitritis, vasculitis, and vascular occlusion was more commonly found in non-HIV patients [13]. However, with the emergence of infectious diseases that are usually seen with compromised immune status in the Middle East and Gulf regions, more data about the clinical pictures and outcomes of these diseases are needed [17]. In this paper, we aim to describe the clinical pictures and long-term outcomes of CMV retinitis in patients with and without HIV infections presenting to a tertiary eye center.

## Methods

This is a retrospective interventional cohort study that included all patients who presented with a clinical picture suggestive of CMV retinitis and had their diagnosis confirmed by polymerase chain reaction (PCR) testing. This study was approved by the Institutional Review Board (IRB) at King Khaled Eye Specialist Hospital (KKESH) with IRB number 25,133-R, and the study adhered to the tenets of the Declaration of Helsinki. The electronic files at KKESH were searched for the terms (cytomegalovirus retinitis) and (CMV retinitis) to search for cases from 2014 to 2024. Cases of acute retinal necrosis (ARN), ocular syphilis disease, and cases that were not confirmed by PCR testing were excluded. The diagnosis of CMV retinitis was based on the classification criteria of CMV retinitis approved by the Executive Committee of the Standardization of Uveitis Nomenclature (SUN) Working Group in 2021 [18], which include the following criteria:

1. Necrotizing retinitis with indistinct borders due to numerous small satellites.
2. Evidence of immune compromise.
3. Either a characteristic clinical appearance or positive polymerase chain reaction assay results for CMV from an intraocular specimen.

A detailed review of the electronic records of all included patients was performed, including detailed immunological history, ophthalmic examination on presentation, PCR test results, systemic and ocular treatments, ocular complications, and final visual outcomes. Multimodal retinal imaging, including fundus photography (Optos PLC, Dunfermline, UK) and spectral domain optical coherence tomography (SD-OCT; Spectralis, Heidelberg engineering, Hiedlberg, Germany), was performed. Poor visual outcome was identified as a final BCVA of 20/200 or worse. The outcomes were reported per patient for the demographics and systemic treatments and per eye for the clinical findings and ophthalmic treatments.

The zones of retinitis were described as zone 1 if retinitis was located within 3000 mm of the center of the fovea (macular involvement) or within 1500 mm of the margin of the optic disc (disc involvement), zone 2 if it was located anteriorly to zone 1 up to the equator, and zone 3 if it was located between zone 2 and ora serrata [8]. The data were analyzed using IBM SPSS Statistics version 26.0 (IBM, Armonk, NY, USA). Frequencies and percentages were computed to describe categorical data. Means with standard deviations were computed to describe continuous data. Fisher’s exact test was used to compare categorical data, and analysis of variance (ANOVA) was used for comparison of numerical data. Penalized regression was performed to analyze the possible risk factors associated with poor visual outcomes. A P-value of < 0.05 at 95% confidence interval (CI) was considered significant.

## Results

**Fig. 1:**
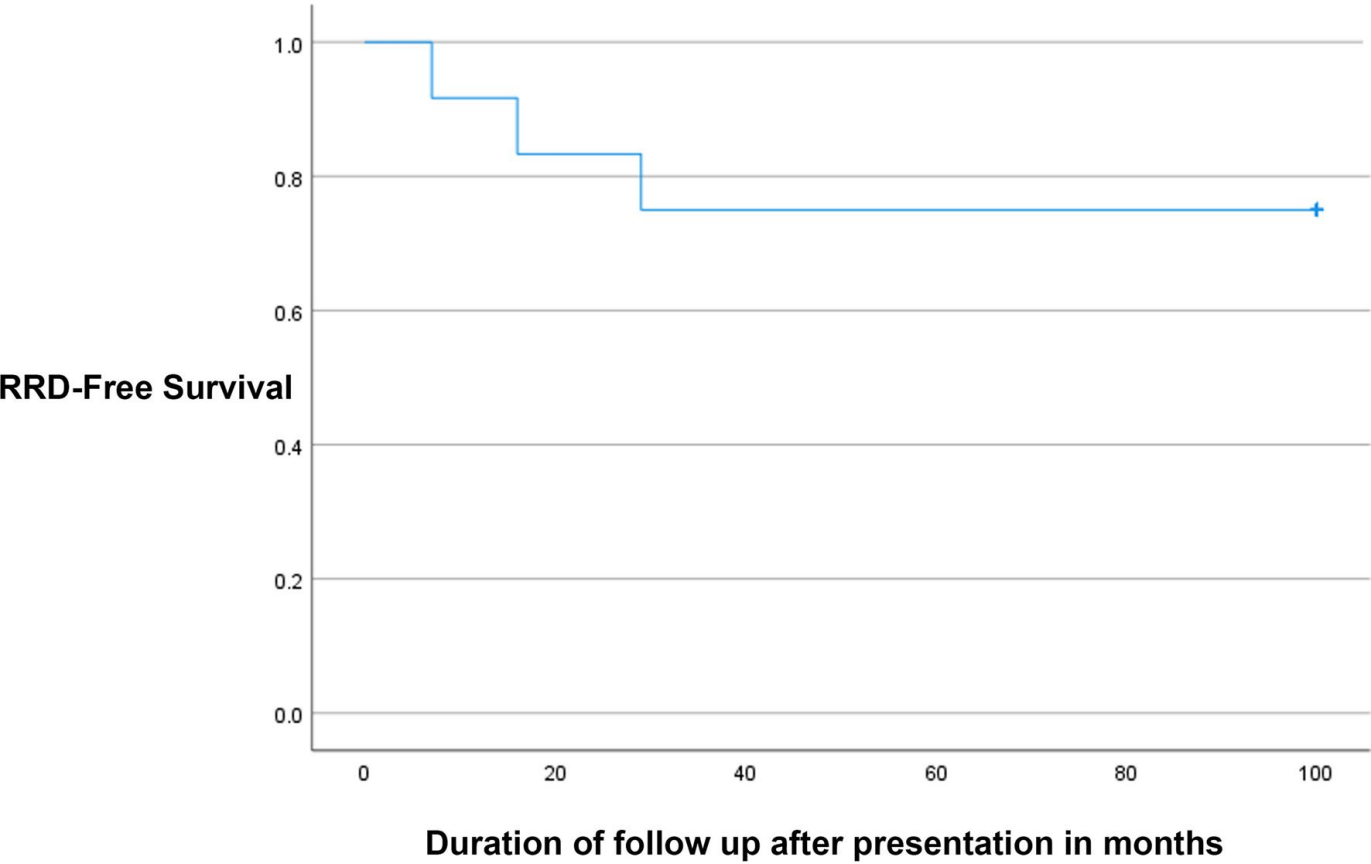
Kaplan Meier survival curve of the probability of cytomegalovirus (CMV) reinitis eyes not developing rhegmatogenous retinal detachments (RRD) over time

**Fig. 2:**
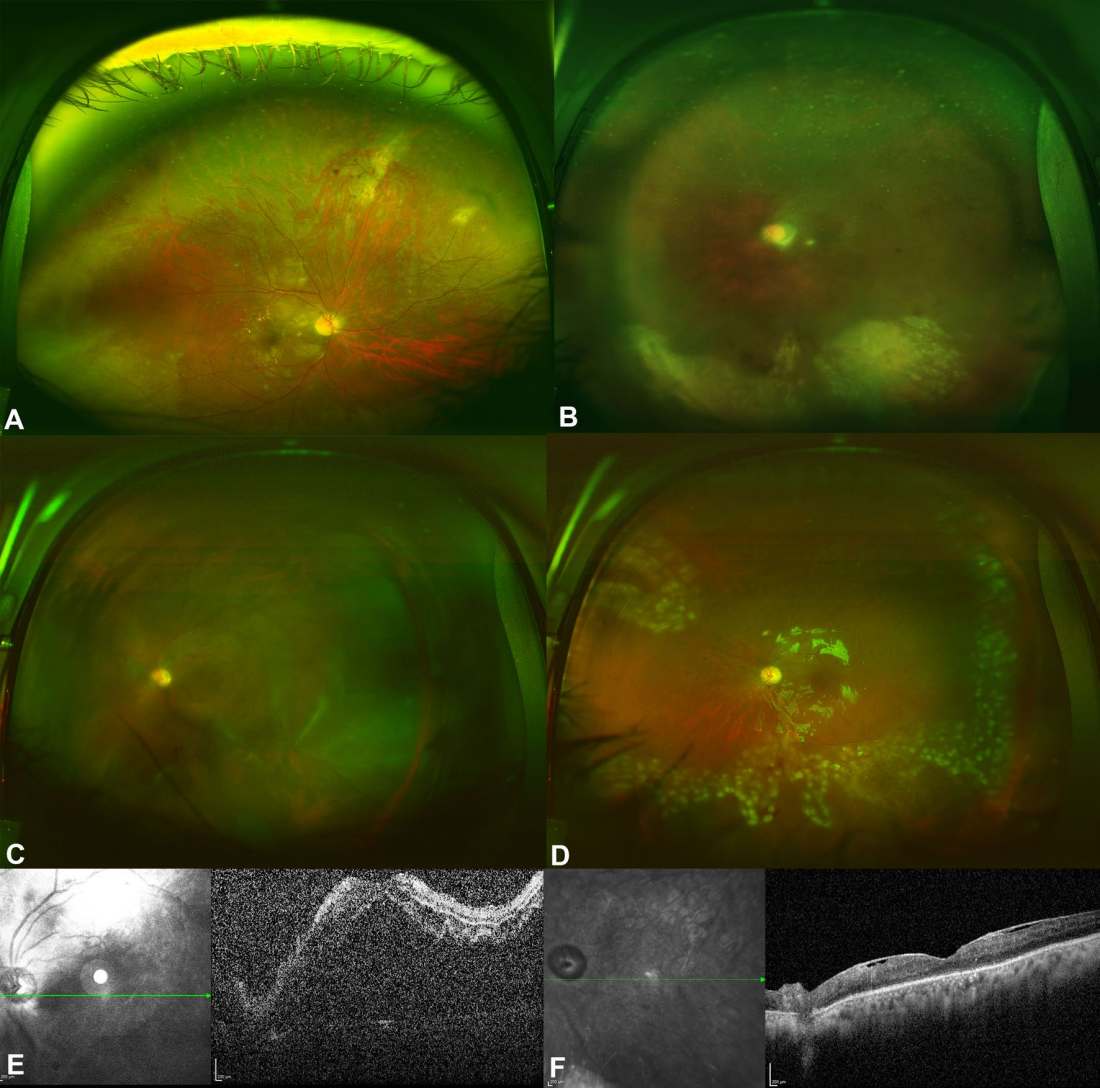
**A** is a color fundus photo of the right eye in a 56-year-old man who developed cytomegalovirus (CMV) retinitis after renal transplantation, showing multiple areas of focal hemorrhagic retinitis in the macula and superonasally, along with diffuse atrophic retinal changes and macular edema. **B** is a color fundus photo of the left eye showing 1 + vitritis and diffuse retinitis infero-temporally and infero-nasally. **C** is a color fundus photo of the left eye showing rhegmatogenous retinal detachment (RRD). **D** is a color fundus photo showing a flat retina after surgical repair with silicone oil reflections. **E** is a spectral domain optical coherence tomography (SD-OCT) photo of the left eye showing a detached macula. **F** is an SD-OCT showing a flat macula with atrophic changes and epiretinal membrane formation

**Fig. 3:**
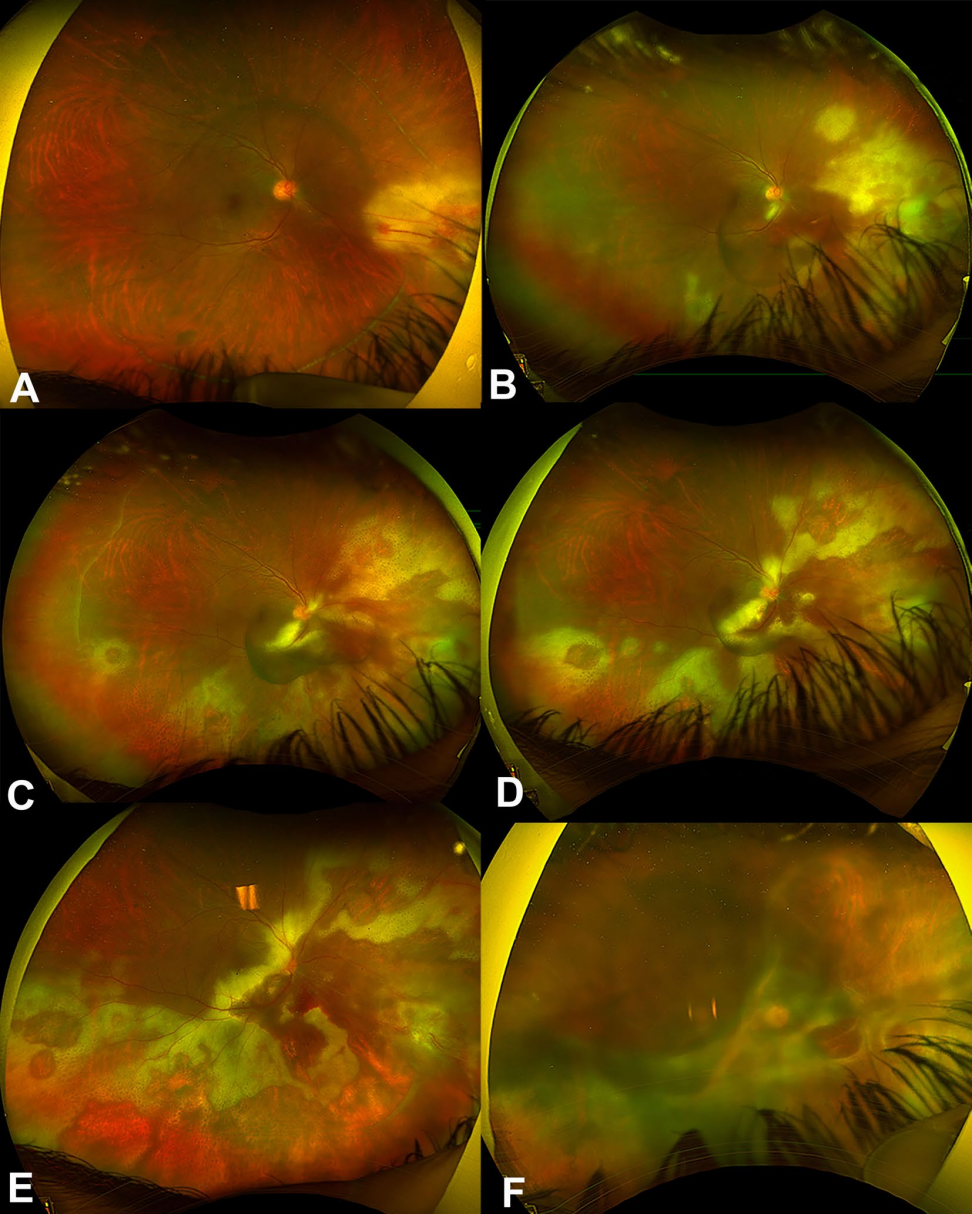
**A** is a color photo of the right eye in a 30-year-old man with human immunodeficiency virus (HIV) infection and developed CMV retinitis showing focal hemorrhagic retinitis nasally. **B** is a color photo of the right eye showing progression of retinitis to involve the supero-nasal, inferonasal, and inferior retina. **C** is a color photo of the right eye showing progression of retinitis with optic disc involvement. **D** is a color photo of the right eye showing more progression and involvement of the optic disc and extension to the inferotemporal retina. **E** is a color photo of the right eye showing more proximal retinal involvement along with the optic disc involvement. **F** is a color photo of the right eye showing a total RRD, which was deemed inoperable

**Fig. 4:**
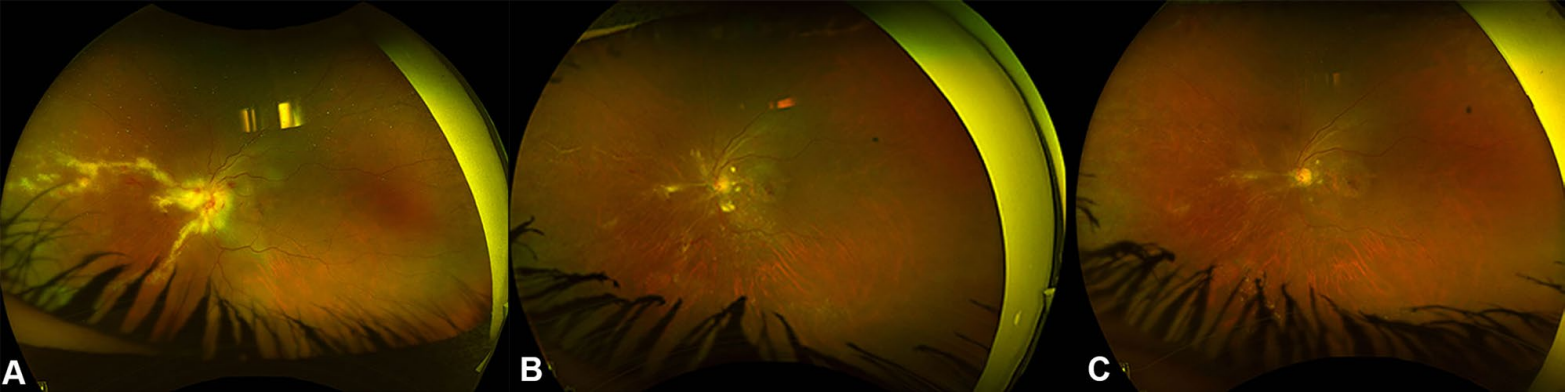
**A** is a color fundus photo of the left eye in a 44-year-old female with HIV infection and developed CMV retinitis showing hemorrhagic retinitis extending from the optic disc towards the nasal retina. **B** is a color fundus photo of the left eye showing consolidation of retinitis, leaving atrophic retinal changes nasally along with sclerosed blood vessels. **C** is a color fundus photo of the left eye showing marked improvement of retinitis and a healthy optic disc

Twelve eyes of 8 patients were included. Five patients (62.5%) were males, and 3 (37.5%) were females. The mean duration of follow-up was 9.2 ± 6.9 years. Four patients (40%) were on immunosuppressive medications after renal transplantations for chronic renal failure (CRF). The reasons for CRF were uncontrolled diabetes and hypertension in 3 patients and lupus nephritis in one patient. One patient had a congenital immunodeficiency disease, and three patients had human immunodeficiency virus (HIV) infections. Three patients were on oral prednisolone, one patient was on mycophenolate mofetil, and one patient was on Tacrolimus. The mean duration of immunosuppressive medications use was 86.5 ± 69.8 months. Four patients (50%) had bilateral involvement, and 4 patients had unilateral involvement. Five eyes (41.7%) were right eyes, and 7 eyes (58.3%) were left eyes. The baseline best-corrected visual acuity (BCVA) was 0.8 ± 0.9 (Snellen = 20/125). Vitritis (Supplementary Fig. 1) was found only in 2 eyes (16.7%). Seven eyes (58.3%) had a hemorrhagic type of retinitis (Supplementary Fig. 2), and 5 eyes had granular retinitis (41.7%). Table 1 shows the clinical examination details of the 12 eyes included in the study. All eyes had positive polymerase chain reaction (PCR) tests for CMV from aqueous samples. All patients were treated with oral valganciclovir 900 mg twice daily for 21 days, followed by once daily. The total duration of treatment with oral valganciclovir was 5.4 ± 4.2 months. Five eyes (41.7%) were treated with intravitreal ganciclovir with an average of 6.4 ± 1.5 injections. All three patients with HIV infections were treated with Highly Active Antiretroviral Therapy (HAART). Optic nerve involvement was observed in two eyes on presentation. In one eye, it was an early involvement that was improved with treatment and resulted in 20/60 vision, while optic involvement was extensive and resulted in total optic disc pallor and no light perception (NLP) vision in one eye (Supplementary Fig. 3). Rhegmatogenous retinal detachment (RRD) developed in 3 eyes (25%) after 7, 16 and 29 months of follow-ups. Figure 1 shows the Kaplan Meier survival curve of the probability of CMV retinitis eyes not developing RRD over time. Two eyes were of HIV patients, and one eye of a post-renal transplantation patient on immunosuppressive medications. The extensions of retinitis were 6 clock hours in two eyes which had granular retinitis, and 4 clock hours in an eye which had a hemorrhagic retinitis. Two RRDs had surgical repairs with pars plana vitrectomy, endolaser, and silicone oil (Fig. 2). One of the operated eyes in a diabetic patient developed proliferative diabetic retinopathy after surgery despite the intraoperative laser application inferiorly and supero-nasally for the corresponding retinal breaks with severe non-proliferative diabetic retinopathy in the contralateral eye and eventually developed neovascular glaucoma (NVG) and was treated with Ahmed Glaucoma valve (AGV) implantation One eye in an HIV patient who was not compliant with HAART treatment had progressive retinitis, which later involved the optic nerve and resulted in an RRD that was deemed inoperable (Fig. 3). In addition, the central nervous system was later involved, and the patient developed encephalitis and ependymitis. Total resolution of retinitis without visual threatening complications was achieved in 6 eyes (50%), including one eye that had an early optic disc involvement (Fig. 4). There were no significant differences between eyes that had HIV infections and eyes that did not have HIV infections. Table 2 shows the comparisons between the eyes of patients with HIV infections and eyes with other causes of immunosuppression. The mean BCVA on the last visit was 0.9 ± 1.2 (Snellen = 20/160), and the mean IOP was 15.1 ± 5.1 mmHg. The change in BCVA was statistically significant (*P* = 0.027). There were marginally insignificant relationships between macular involvement of retinitis (*P* = 0.091) and the incidence of RRD (*P* = 0.091) with poor visual outcomes. Table 3 shows the relationship analysis between several factors and poor visual outcomes.

**Table 1:** The clinical features of 12 eyes in 8 patients with cytomegalovirus retinitis

Clinical Feature	Number of eyes (%)
Best-corrected visual acuity	0.8 ± 0.9 (Snellen = 20/125)
Intraocular pressure	15.17 ± 4.5 mmHg
Keratic precipitates	Yes 3 (25) No 9 (75)
Anterior chamber	Quite 8 (66.7) Trace cells 3 (25) 1 + cells 1 (8.3)
Lens	Clear 9 (75) Posterior chamber intraocular lens 2 (16.7) Cataract 1 (8.3)
Vitritis	Yes 2 (16.7) No 11 (83.3)
Type of retinitis	Hemorrhagic 7 (58.3) Granular 5 (41.7)
Extent of retinitis	2.8 ± 1.7 clock hours
Area of retinitis	Supero-nasal 6 (50) Infero-nasal 5 (41.7) Infero-temporal 5 (41.7) Superotemporal 2 (16.7)
Zone of retinitis	Zone I 5 (41.7) Zone II 9 (75.0) Zone III 6 (50.0)
Optic disc involvement	Yes 2 (16.7) No 10 (83.3)
Macular Involvement	Yes 3 (25) No 9 (75)
Vasculitis	Yes 3 (25) No 9 (75)
Vascular occlusion	Yes 2 (16.7) No 10 (83.3)
Macular optical coherence tomography	Normal 5 (41.7) Cystoid macular edema 2 (16.7) Atrophic 2 (16.7) Detached 1 (8.3) Not available 2 (16.7)

**Table 2:** The comparisons between eyes of patients with HIV infections and eyes with other causes of immunosuppression

Feature	Eyes with HIV (4)	Eyes without HIV (8)	Significance (95% CI)	Odds Ratio (95% CI)
Age	43.5 ± 9.4 years	51.0 ± 14.1 years	*P* = 0.363	N/A
Laterality	Unilateral 2 Bilateral 2	Unilateral 2 Bilateral 6	*P* = 0.406	2.0
BCVA	0.58 ± 0.52 (20/80)	0.8 ± 1.0 (20/125)	*P* = 0.640	N/A
IOP	15.0 ± 5.5	15.3 ± 4.4	*P* = 0.933	N/A
KPs	Yes 0 No 4	Yes 3 No 5	*P* = 0.255	1.6
AC reaction	Quite 3 Trace 1 1 + 0	Quite 5 Trace 2 1 + 1	*P* = 0.755	N/A
Lens	Clear 4 PCIOL 0 Cataract 0	Clear 5 PCIOL 2 Cataract 1	*P* = 0.368	N/A
Vitritis	No 4 Yes 0	No 7 Yes 2	*P* = 0.424	2.0
Macular involvement	Yes 1 No 3	Yes 2 No 6	*P* = 0.764	1.0
Optic disc involvement	Yes 1 No 3	Yes 1 No 7	*P* = 0.576	2.0
Vasculitis	Yes 0 No 4	Yes 3 No 5	*P* = 0.255	2.7
Type of retinitis	Hemorrhagic 2 Granular 2	Hemorrhagic 5 Granular 3	*P* = 0.576	0.8
Zone of retinitis	Zone I 2 Zone II 4 Zone III 1	Zone I 3 Zone II 5 Zone III 5	*P* = 0.319	N/A
Extension of retinitis	2.0 ± 3.0 clock hours	2.6 ± 1.7 clock hours	*P* = 0.739	N/A
Vascular occlusion	Yes 0 No 4	Yes 2 No 6	*P* = 0.424	1.3
RRD	Yes 2 No 2	Yes 1 No 7	*P* = 0.236	4.0
Glaucoma	Yes 0 No 4	Yes 1 No 7	*P* = 0.667	1.1
Final outcome	Good 2 Poor 2	Yes 4 No 4	*P* = 0.727	1.0
Final BCVA	1.4 ± 1.5 (20/500)	0.7 ± 1.0 (20/100)	*P* = 0.365	N/A
Final IOP	12.3 ± 6.9 mmHg	16.5 ± 3.7 mmHg	*P* = 0.184	N/A

AC: Anterior chamber; BCVA: best-corrected visual acuity; HIV: human immunodeficiency virus; IOP: intraocular pressure; KPs: keratic precipitates; PCIOL: Posterior chamber intraocular lens; RRD: rhegmatogenous retinal detachments

**Table 3:** Analysis of several factors in relation to the final visual outcomes

Factor	Significance (95% CI)	Odds Ratio (95% CI)
Vitritis	*P* = 0.773	1.0
Area of retinitis	*P* = 0.716	N/A
Zone of retinitis	*P* = 0.284	N/A
Extent of retinitis	*P* = 0.285	N/A
Optic disc involvement	*P* = 0.773	1.0
Macular involvement	*P* = 0.091	3.0
Vasculitis	*P* = 0.500	1.5
Type of retinitis	*P* = 0.500	1.4
Occlusive vasculitis	*P* = 0.773	1.0
RRD	*P* = 0.091	3.0

CI: confidence interval; N/A: not applicable; RRD: rhegmatogenous retinal detachment

## **Discussion**

In this paper, we describe the clinical features and management outcomes of CMV retinitis in a tertiary eye center. In the HIV era, care needs to be taken not to misdiagnose or delay the diagnosis of this vision-threatening disease [16, 17, 19]. Previous studies suggested that, unlike HIV infected patients, the clinical picture of CMV retinitis in non-HIV retinitis patients more commonly involves vitritis, vasculitis, and occlusive vasculitis, leaning towards the clinical picture of acute retinal necrosis (ARN) commonly caused by herpes simplex and herpes zoster viruses [13, 15, 16]. This study also shows that vitritis and vasculitis were found in the non-HIV subset of patients and were absent in HIV infected patients. This might be related to the fact that CMV can potentiate the pathogenicity of HIV by activating HIV, increasing the intracellular transport of HIV, and affecting CD8 T cell function. CMV also increases HIV viral replication, further decreasing the immunity of the hosts [20]. On the other hand, some reports suggested that patients with non-HIV infection-related CMV retinitis had quiet eyes with absence of vitritis and anterior chamber reactions [11].

In our experience, we found that the term (non-HIV) is a generalized term that lumps multiple causes of immunocompromise together. Patients who had renal transplantations in this series developed vitritis and anterior chamber reactions, while vasculitis was present in a patient who had renal transplantation and a patient who had a severe congenital immune deficiency, and ended up with occlusive vasculitis in the latter patient. Notably, patients with non-HIV-infected CMV retinitis presented with lower visual acuities, larger extent of retinitis, and higher risks of macular involvements. These findings were similar to previous findings in these patients [12]. There were fewer zone I retinitis involvements and incidences of vasculitis in non-HIV infected eyes. The differences in clinical features between HIV-infected and non-HIV infected eyes were not statistically significant. These results might be explained by the small number of eyes in both groups and the heterogeneity of the causes of compromised immunity in these patients, making the elimination of type II error less certain .

The overall incidence of RRD in our patients was 25% and was slightly higher among patients with HIV-related CMV retinitis. This is slightly higher than previously reported rates of RRD in CMV retinitis and might be explained by the larger extent of retinitis leading to RRD [11, 15, 21]. The higher risk of RRD in eyes with a larger extent of retinitis was also found in ARN and was more commonly seen in eyes with multifocal or diffuse retinitis [22]. However, unlike ARN, which had an increased risk of RRD with higher grades of vitritis, RRD in CMV retinitis was not related to vitritis [22]. This might be explained by the low grades of vitritis in CMV retinitis, which result in minimal inflammatory changes that contribute to the mechanism of RRD. Nevertheless, the inflammatory component in RRD formation is an important contributor, as the risk of RRD in ARN eyes is much higher (56%) than in eyes with CMV retinitis.

Other complications that affected visual outcomes in this study include retinal atrophic changes from proximal involvement of retinitis, optic nerve involvement, and NVG. Previous studies have also reported these complications in CMV retinitis [11, 16, 23]. NVG is thought to be caused by peripheral retinal ischemia induced by a large area of retinitis [16]. In our study, one eye of a diabetic patient had NVG after undergoing a surgical repair for RRD in which laser was applied to the areas of retinitis, indicating that the development of NVG was probably aggravated by the peripheral inflammation-induced retinal ischemia, in addition to diabetic retinopathy, as the diabetic retinopathy changes were non-proliferative and less prominent in the contralateral eye. Appropriate and timely laser photocoagulation application to the ischemic retina, especially in patients with concurrent diabetes helps avoid the ischemia-related complications of NVG and vitreous hemorrhage.

Optic nerve involvement was observed in 2 eyes of 2 patients: One HIV infected patient and one with severe congenital immunodeficiency, resulting in total optic disc pallor in one eye and good visual recovery in response to HAART and systemic and local treatment in one eye. The importance of early recognition of optic disc involvement in CMV retinitis cannot be overemphasized. Another condition that needs to be differentiated from optic nerve involvement in CMV retinitis is neuroretinitis, either infectious or non-infectious. In addition to the classical picture of hemorrhagic or granular retinitis of CMV infection, neuroretinitis usually involves more prominent vitreous involvement in the form of prepapillary vitreous opacities in non-infectious neuroretinitis and higher grades of focal or diffuse vitritis in toxoplasmosis-related neuroretinitis and macular exudation in cat-scratch disease [24].

Macular involvement was noted in 3 eyes and affected the visual outcomes. Poor visual outcomes as a result of macular involvement were also observed in previous studies [12]. The reason for these outcomes is the retinal atrophic changes, which persist even after the administration of proper treatments.

All of our patients were treated with Valganciclovir, and intravitreal ganciclovir was administered to 41% of eyes in our patients. The reasons for not giving intravitreal injections in the remaining eyes included patient refusal, limited availability, and total visual loss beyond salvation. In addition, HAART constitutes an integral part of management in patients infected with HIV. The non-compliance with the treatment in one patient resulted in more extensive CMV retinitis and eventually inoperable RRD, leading to total visual loss as the other eye was lost before presentation.

It is important to have a high index of suspicion for CMV in patients who present with retinitis with low-grade inflammations, especially in the presence of an immunocompromised status. Early administration of intravitreal ganciclovir or foscarnet, along with systemic antiviral therapy, might hasten control of CMV infection and prevent further visual compromise. Close monitoring and serial clinical evaluations are important to check for response to treatments, adherence to antiviral maintenance therapy, and assess for the risks of retinal detachments. Establishing a multidisciplinary care, which includes infectious disease and immunology teams is considered the cornerstone of management of patients with CMV infections.

This study has several limitations, including the retrospective nature, the differences in clinical assessments, investigations, and treatment regimens between the treating physicians, and small patient numbers. In addition, the unavailability of hematologic and immunologic evaluations, including CD4 counts, plasma CMV load and HAART treatment data due to the management of these cases in other facilities which provide further infectious and immunological evaluations are further limitations to this study. Importantly, there are several factors that contributed to the limited number of patients. These factors include patient-related social and cultural barriers, limited access to tertiary eye care for patients who are debilitated or severely immunocompromised, and the loss to follow-up in some patients, preventing the assessment of treatment efficacy. Despite these limitations, the current study provides real-world data regarding the clinical features of patients with and without HIV infection in a region where data about this disease are lacking, and adds to the body of evidence on long-term outcomes of CMV retinitis.

In conclusion, the clinical features of CMV retinitis are more accurately related to the level of immunity than to the classification of HIV vs. non-HIV CMV retinitis. The absence of vitritis, vasculitis, and occlusive vasculitis is an important feature of HIV-related CMV retinitis. This indicates an absence of inflammatory reaction and an inability to fight the opportunistic viral infection. Timely recognition and appropriate treatment are critical to achieve better long-term visual recovery and avoid vision-threatening complications like RRD, macular atrophy, optic nerve involvement, and NVG.

## Supplementary Information

Below is the link to the electronic supplementary material.

Supplementary Material 1: Fig. 1: A is a color fundus photo of the left eye in a 73-year-old woman who developed cytomegalovirus (CMV) retinitis after renal transplantation, showing vitritis and supero-nasal granular retinitis. B is a color fundus photo of the left eye showing resolved retinitis and improved vitritis.

Supplementary Material 2: Fig. 2: A is a color fundus photo of the left eye in a 27-year-old woman after renal transplantation for lupus nephritis and developed CMV retinitis, showing superior and superotemporal hemorrhagic retinitis. B is a color fundus photo of the left eye showing resolved retinitis leaving atrophic retinal changes.

Supplementary Material 3: Fig. 3: A is a color fundus photo of the left eye in a 41-year-old man with severe congenital immunodeficiency and developed CMV retinitis showing diffuse hemorrhagic retinitis involving the macula and optic disc. B is a color fundus photo of the left eye showing a pale disc and an atrophic retina.

## Data Availability

All data generated or analyzed during this study are included in this article. Further enquiries can be directed to the corresponding author.
