# Supplementary figures and images for: CMV retinitis: the diagnostic challenges and long-term outcomes. The experience of tertiary eye center in Saudi Arabia
Source: J Ophthalmic Inflamm Infect. 2026 Mar 11;16:16. doi: 10.1186/s12348-026-00572-3 (PMC13087059; doi:10.1186/s12348-026-00572-3)

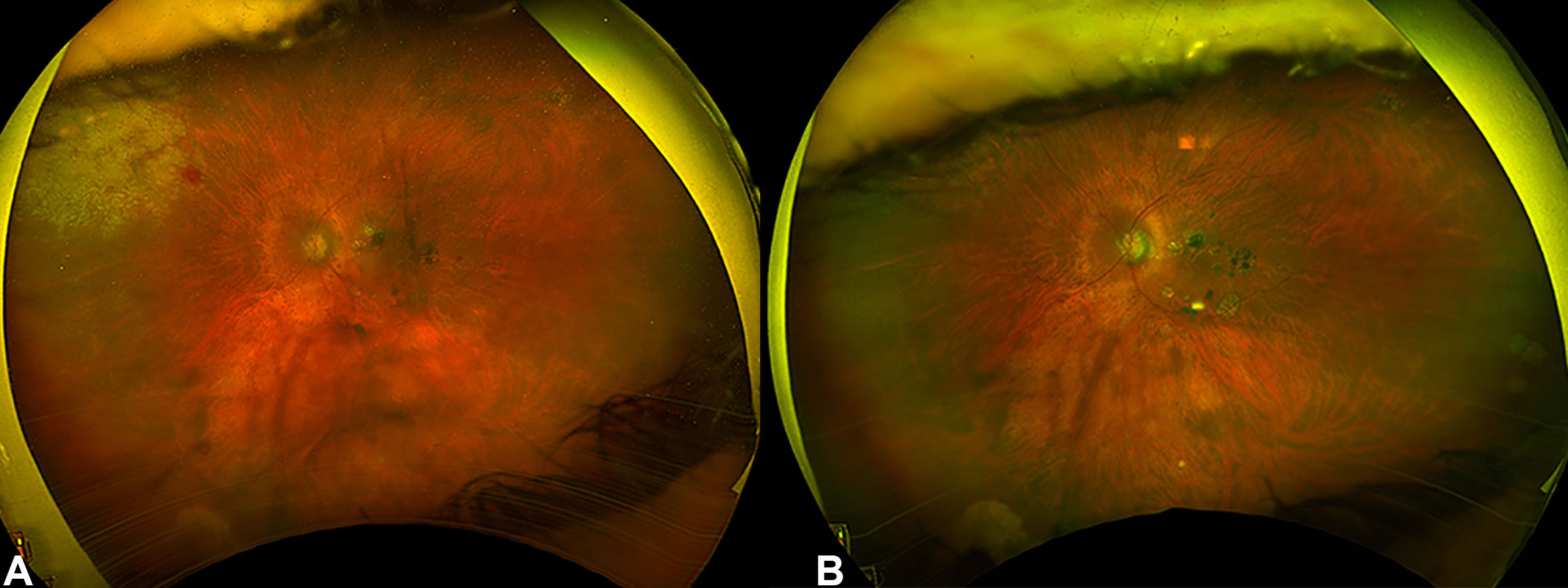

Supplement: Supplementary file 1 — Supplementary Material 1: Fig. 1: A is a color fundus photo of the left eye in a 73-year-old woman who developed cytomegalovirus (CMV) retinitis after renal transplantation, showing vitritis and supero-nasal granular retinitis. B is a color fundus photo of the left eye showing resolved retinitis and improved vitritis. [file 12348_2026_572_MOESM1_ESM.jpg]

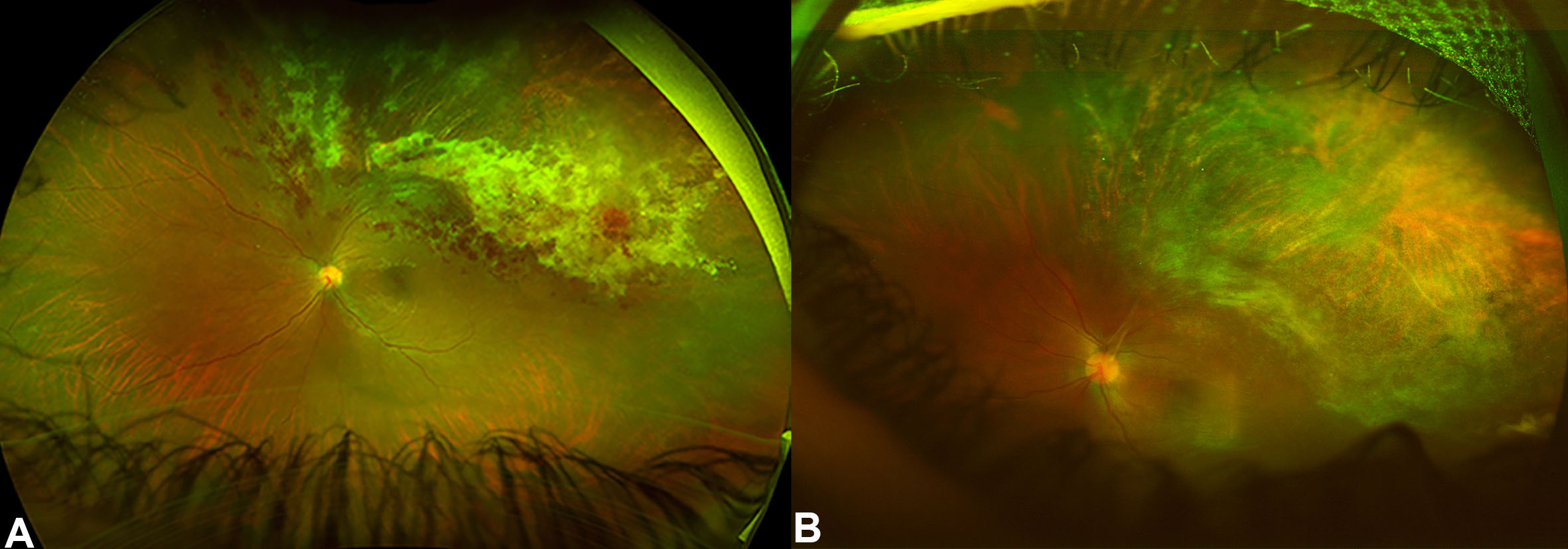

Supplement: Supplementary file 2 — Supplementary Material 2: Fig. 2: A is a color fundus photo of the left eye in a 27-year-old woman after renal transplantation for lupus nephritis and developed CMV retinitis, showing superior and superotemporal hemorrhagic retinitis. B is a color fundus photo of the left eye showing resolved retinitis leaving atrophic retinal changes. [file 12348_2026_572_MOESM2_ESM.jpg]

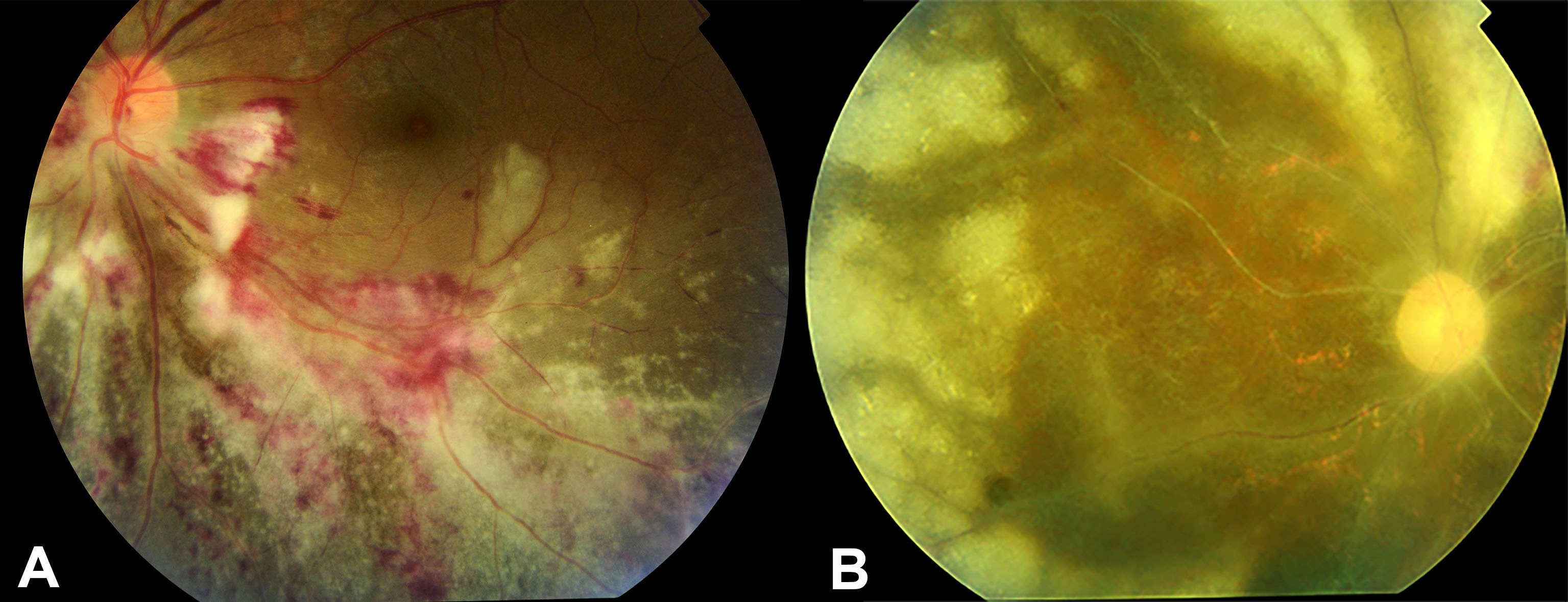

Supplement: Supplementary file 3 — Supplementary Material 3: Fig. 3: A is a color fundus photo of the left eye in a 41-year-old man with severe congenital immunodeficiency and developed CMV retinitis showing diffuse hemorrhagic retinitis involving the macula and optic disc. B is a color fundus photo of the left eye showing a pale disc and an atrophic retina. [file 12348_2026_572_MOESM3_ESM.jpg]
